# Upper Limb Neuromuscular Activities and Synergies Comparison between Elite and Nonelite Athletics in Badminton Overhead Forehand Smash

**DOI:** 10.1155/2018/6067807

**Published:** 2018-12-20

**Authors:** Hamidreza Barnamehei, Farhad Tabatabai Ghomsheh, Afsaneh Safar Cherati, Majid Pouladian

**Affiliations:** ^1^Department of Biomedical Engineering, Science and Research Branch, Islamic Azad University, Tehran, Iran; ^2^Pediatric Neurorehabilitation Research Center University of Social Welfare and Rehabilitation Sciences, Tehran, Iran; ^3^Iran university of Medical Science, Rasoul Akram Hospital, Tehran, Iran

## Abstract

This study is aimed at comparing muscle activations and synergies in badminton forehand overhead smash (BFOS) between elite and nonelite players to clarify how the central nervous system (CNS) controls neuromuscular synergy and activation to generate complex overhead movements. EMG of five upper limb muscles was recorded through surface electromyography (EMG) electrodes from twenty players. Athletics is divided into two groups: elite and nonelite. Eventually, nonnegative matrix factorization (NNMF) was utilized to the calculated electromyography signals for muscle synergy comparison. Similarities between elite and nonelite groups were calculated by scalar product method. Results presented that three muscles synergies could sufficiently delineate the found electromyography signals for elite and nonelite players. Individual muscle patterns were moderately to highly similar between elite and nonelite groups (between-group similarity range: 0.52 to 0.90). In addition, high similarities between groups were found for the shape of synergy activation coefficients (range: 0.85 to 0.89). These results indicate that the synergistic organization of muscle coordination during badminton forehand overhead smash is not profoundly affected by expertise.

## 1. Introduction

The badminton forehand overhead smash (BFOS) is one of the high speed and powerful motions among various racket sports [1]. Badminton is a sport that requires a lot of overhead shoulder movement, with the shoulder in abduction and external rotation and generally proximal-to-distal sequence [2, 3]. However, it appears that the proximal-to-distal sequencing may be inadequate to accurately describe some shoulder complex motions as overhead tasks [4]. Among overhead athletes, badminton is well known as a relatively low-risk injury compared to other sports and the risk of shoulder injury may be increased with the style of stroke, the length of the throw, the level of athlete, and the level of associated muscle fatigue that happens during training [5]. A higher level of activation in the overhead task was observed with the velocity increase in the external oblique, latissimus dorsi, middle deltoid, biceps brachii, and triceps brachii [6]. The shoulder muscles must control two motions: to generate powerful moment for motion and to keep the integrity of the shoulder joint complex [7]. The dynamic motion of the overhead task relies on the interaction of a series of structural and functional components of the neuromuscular system [8].

One of the proposed hypotheses to understand motor control is the muscle synergy concept [9]. Muscle synergy is a strategy of the central nervous system (CNS) to reduce the redundancy at the musculoskeletal level [7, 10]. The control of movements by the CNS is made by interpreting the task-level commands into a reduced number of synergies [11]. Muscle synergy is hypothesized to be the method by which central processing of human body simplifies motor control, and synergistic muscular activation patterns have been observed while acting particular motion, e.g., overhead motions. Muscles synergies, that are groupings of functionally similar muscle, have been found in humans across a wide diversity of motions such as reaching [12], grasping [13], walking and running [14], postural response [15], and isometric force generation [16]. It has also been observed that the synergies are shared across different tasks [17].

One of the crucial determinants for the performance is the ability of the badminton players to coordinate the upper limbs, particularly during the overhead motions. Therefore, comprehensive information about coordination strategies during badminton overhead smash may benefit athletes and coaches. In addition, the recognition of muscle synergies and EMG patterns in elite and nonelite players during overhead sports can characterize the motor control patterns and neuromuscular coordination promoted with training [18, 19]. The identification of muscle recruitment and muscle synergy patterns in experienced athletes can characterize the skill patterns and neuromuscular coordination developed with training. Thus, this characterization could be an important factor to the players, coaches, and sports medicine to define training strategies that lead to superior performance, to avoid learning faults and prevent feasible injuries that happen with some frequency in the upper extremities [4]. Moreover, despite the domination of the overhead motions in racket sports, there is a lack of information on upper limb neuromuscular activation patterns and therefore on the coordination among upper limb muscles. Eventually, the comparison between elite and nonelite players is scarce. Though there is no effect of expertise on neuromuscular coordination during basic motor tasks such as bench press [13] or rowing [20], it does not elucidate whether a more complex motor task such as badminton forehand overhead smash is associated with different muscle coordination strategies between elite and nonelite badminton players.

Therefore, the aim of this study was to compare the muscle activation and muscle synergies of BFOS between elite and nonelite groups to find motor strategy discrepancies between groups for the recognition of neuromuscular synergies and coordination. So, the novelty of the present study is muscle synergy comparison of the shoulder complex between elite and nonelite badminton players to finding CNS discrepancies. In addition, recognition of shoulder muscle synergies in a different phase of high-speed overhead tasks in elite and nonelite players can provide information about how muscle coordination varies in different phase and skill levels. We hypothesized that the muscle synergies would be different between groups due to differences of a training plan and nonelite players may improve if they get the synergy as elite players. If we could clarify the factor to improve the performance using muscle synergy method in the current study, muscle synergy analysis may help not only badminton but also any other overhead motion the plan of training program to improve the performance. By filling this gap in the literature, we will have better insights regarding motor control in sports which will specifically improve high-speed overhead motion training [21].

## 2. Materials and Methods

### 2.1. Subjects

Twenty volunteers were divided in two groups in the study; that composed eight men and four women from the Iran badminton national team, all elite players with mean practice of 15 years and more than 4 years of national and international competitive experience, and a control group of four men and four women without previous professional experience in badminton but play badminton for entertainment. All participants signed an informed consent document approved by the Faculty of Biomedical Engineering, University of SRBIAU ethical committee. The physical characteristics of the participants are shown in Table 1.

### 2.2. Experimental Procedure

**Figure 1:**
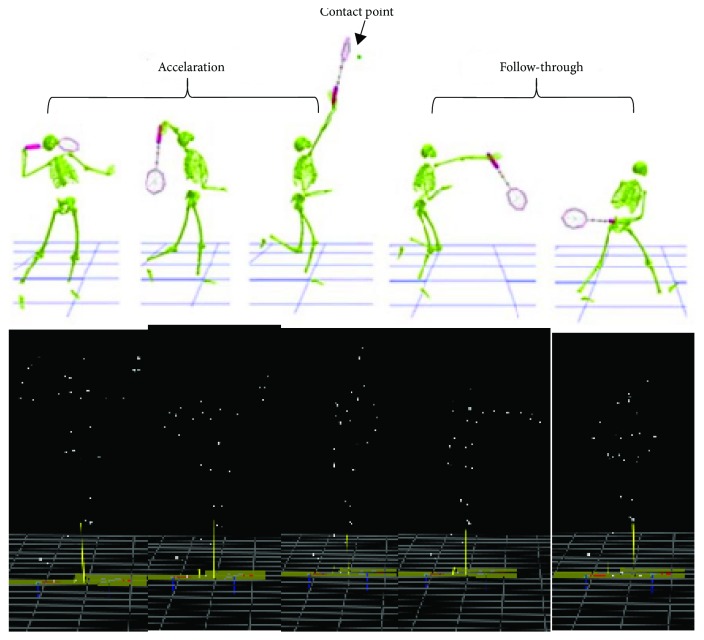
The different phase of badminton forehand overhead smash (BFOS): acceleration phase (before contact point), follow through (after contact point).

The experimental study focused on the biomechanical analysis of a badminton forehand overhead smash (BFOS) movement performed by the two different badminton groups, the elite and the nonelite. The BFOS was established by all subjects starting from a static posture. The right upper limb was located with the arm extended at the shoulder joint less than 40 degrees, and at the elbow joint, the forearm was flexed at approximately 90 degrees. Each stroke was analyzed during the interval delimited from the time instant when the elbow joints flex, and the shoulder joints abduct to the time instant where the elbow marker reached the minimum height displacement using video data (Figure 1). The BFOS motion was time normalized and represented on a 0–100% scale. For timing normalization across trials, an envelope of electromyography signals in the BFOS for muscle synergy analysis was created by resampling at 1% of the BFOS to obtain 100 samples. The amplitudes in an envelope were normalized by the maximum voluntary contraction (MVC) in the five muscles.

Figure 1 shows the different phase of BFOS that includes acceleration phase (before contact) and follow-through phase (after contact). Each subject performed five repetitions of the overhead smash, with a rest period of 5 min between repetitions.

### 2.3. Motion Analysis

All data collection took part in the biomechanics laboratory at the University of Sharif (research center of Movafaghian). For kinematic analysis, 39 markers were placed on the athletes' bodies according to the VICON Plug-in Gait marker Placement. The smaller marker size was chosen to minimize disruption in the overhead task. The BFOS was recorded at 200 Hz by six VICON (Vicon Industries Ltd., Hampshire, United Kingdom) and small reflective markers and analyzed by the VICON Nexus 2.0 software. The angular velocity and acceleration of elbow and shoulder joints have been calculated by the MATLAB software using the angular position data to calculate by derivation of the angular position during the trials.

### 2.4. EMG

The muscle activity of five muscles of the upper limb was recorded: the middle deltoid (DL) muscle, the infraspinatus (IS) muscle, the biceps brachii (BB) muscle, the supraspinatus (SS) muscle, and the serratus anterior (SA) muscle. The surface EMG recordings were made using self-adhesive with Ag/Agcl. The diameter of each area of conductivity was 10 mm while the distance between electrodes was 20 mm. After skin preparation and skin shaving and cleaning with alcohol were done to minimize resistivity before the electrodes installing, electrodes were placed in the center of each muscular belly in a longitudinal orientation. Fine placement of electrodes was required to accurately record neuromuscular bioelectric signals from the rotator cuff muscles. The EMG data acquisition was done with a sampling frequency of 1000 Hz and sent to a computer in the lab. The EMG signal processing was performed in MATLAB software. Raw EMG signals were digitally filtered (10–400 Hz), and the full wave was rectified and smoothed by with a low-pass filter of 8 Hz (Butterworth, 4th order). The data analysis was focused on the following variables: the muscle activities and the peak EMG signal amplitude of the upper limb muscle groups during the different BFOS movement phases.

**Figure 2:**
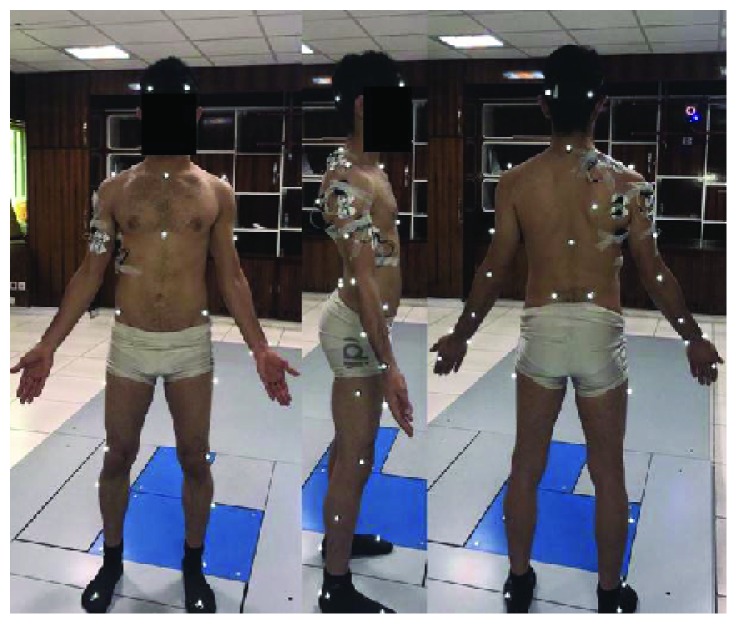
Experimental setup and 39 reflect markers and EMG electrodes were placed on the subject's bodies.

Electromyography signals were normalized using electromyography signals from a maximal voluntary contraction (MVC) as a reference. Three different experiments for MVC were executed for each separate muscle, with 1 min rest between examinations. The experiments were performed with the segments positioned in that each muscle has an intervention as preferential agonist developing its maximal intensity of activation [4]. The kinematic and electromyography signals were synchronized by Nexus software. The whole devices were placed at the end of the warm-up.  Figure 2 shows experimental setup and 39 reflect markers and EMG electrodes were placed on the subject's bodies.

### 2.5. Muscle Synergy

Neuromuscular activity (EMG) signal*s M(*t) recorded the experimental electromyography data and were arranged to form an *D* × *T* matrix *M*, where *D* denotes the number of muscles and *T* denotes the number of samples. Neuromuscular activity (EMG) data *M* was then factorized by NMF to obtain the muscle synergy matrix *W* and the activation coefficient matrix *C*. Matrix factorization minimizes the residual Frobenius norm between the initial matrix and its decomposition, given as follows:

(1) $$\begin{array}{c} M_{D\times T}=W_{D\times N}C_{N\times T}+E_{D\times T},\\ \begin{array}{c} \min{\left\|M-WC\right\|}_{FRO,}\\ W\geq 0,C\geq 0, \end{array} \end{array}$$

where M is the D × T_time_ matrix containing the recorded muscle patterns, W is the D × N muscle synergy matrix, C is the N × T_time_ combination coefficient matrix, and E is the D × T_time_ residual error matrix.

An NNMF (nonnegative matrix factorization) algorithm (Lee and Seung) [19] was used to calculate a set of muscle synergies and their corresponding combination coefficients from the recorded neuromuscular activity.

Different methods have been applied to calculate the number of muscle synergies underlying a given dataset [22–24].

To avoid local minima, in all our subjects, we repeated the analysis by varying the number of synergies between 1 and 10 for each BFOS of each subject, and then, to finalize the number of synergies, we selected the least number of synergies that accounted for >90% of the variance accounted for (VAF) [10, 25, 26]. Global VAF was defined as follows [25]:

(2) $$\begin{array}{c} {VAF}_{Global}=1-\frac{\sum_{i=1}^{p}\sum_{j=1}^{n}{\left(e_{i,j}\right)}^{2}}{\sum_{i=1}^{p}\sum_{j=1}^{n}{\left(E_{i,j}\right)}^{2}}. \end{array}$$

According to Hug et al. [26], we calculated local VAF for each muscle to ensure that each muscle activity pattern was well accounted for by the extracted muscle synergies. We defined the adoption standard that local (for each muscle) VAF > 0.75 [25]. VAF was determined as the uncentered Pearson correlation coefficient. Each vector of muscle activation was compared with its reconstruction as

(3) $$\begin{array}{c} {VAF}_{Local\left(muscle\right)}=1-\frac{\sum_{j=1}^{n}{\left(e_{m,j}\right)}^{2}}{\sum_{j=1}^{n}{\left(E_{m,j}\right)}^{2}}. \end{array}$$

In these equations, *i* goes from 1 to n (n assume a value from 1 to *T*, where *T* is the number of time points) and *m* is the number of muscles (*m* assumes a value from 1 to *D*, where *D* is the number of muscles).

### 2.6. Quantifying Similarity of Synergies

To compare muscle synergies between elite and nonelite athletics, the scalar product which is the similarity index between two synergies was quantified based on a study by Cheung et al. such that [27]

(4) $$\begin{array}{c} Scalar\;Product=\frac{\vec{W_{Elite}}\times \vec{W_{Nonelite}}}{\vec{\left|W_{Elite}\right|}\left|\vec{W_{Nonelite}}\right|},\;0\leq Scalar\;Product\leq 1. \end{array}$$

We defined muscle synergies as the same muscle synergy when scalar product was greater than 0.75 [27]. Furthermore, we quantified the intrareliability and intragroup similarity of synergy using scalar product method. We followed the methods of previous study of Matsunaga et al. [21, 28].

### 2.7. Statistics

Normality was verified through the Shapiro–Wilk test. Therefore, values are reported as means ± standard deviation. Pearson's correlation coefficient (*r*) was used as a similarity criterion for the muscle synergy vectors. As performed in previous studies [20, 26]. All statistical tests were computed using MATLAB 7.8 (MathWorks, Natick, MA) with a significance level of *P* < 0.05.

## 3. Results

**Figure 3:**
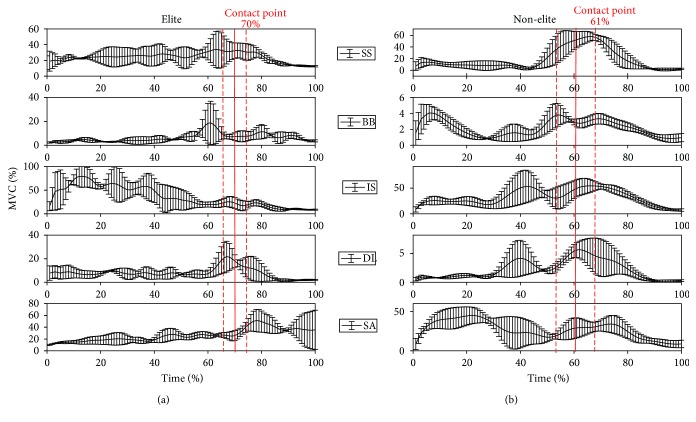
Mean muscle activity (MVC %) and standard deviation for supraspinatus muscle (SS), biceps brachii muscle (BB), infraspinatus muscle (IS), middle deltoid muscle (DL), and serratus anterior muscle (SA) for (a) elite and (b) nonelite groups are shown. Red line and red dash line present the mean contact point and standard deviation.

Figure 3 presents a comparison of five average muscle activities (MVC) between elite and nonelite subjects with contact point and standard deviations.

**Figure 4:**
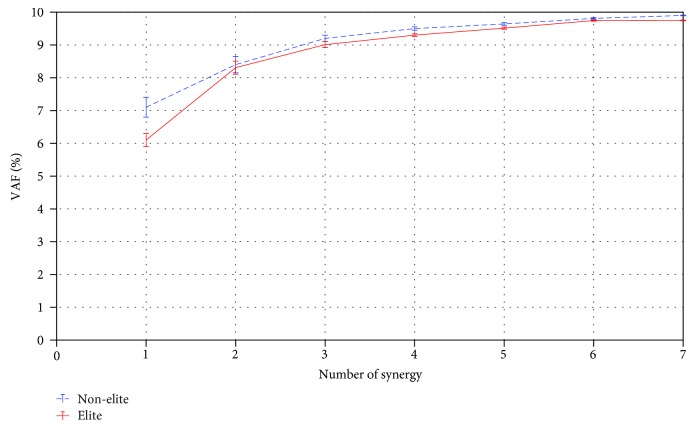
The relationship between the number of synergies and mean VAF (%) after applying synergy analysis with standard deviations for elite and nonelite subjects.

The calculated synergies were used to reconstruct the five upper limb muscle patterns during the BFOS using muscle synergy equation.  Figure 4 presents the cumulative percentages of variance explained by each synergy for each group, and Table 2 presents the local VAF for each muscle when three synergies were identified for both elite and nonelite groups. From these results, three synergies were extracted from the elite athletics (VAF: 0.90 ± 0.01) and three synergies were extracted from the nonelite athletics (VAF: 0.92 ± 0.02). The performance of reconstruction increases with the number of synergies, although, after three synergies for elite and nonelite groups, there is no remarkable change in performance.

Table 3 presents the mean value of scalar product for 3 trials in each subject for elite and nonelite groups. This result represented that there was no mistake for intrareliability in the current study and represented that intragroup reliability was high.

Table 4 shows the mean value of scalar product that was used to indicate similarity of synergy and muscles. The scalar product that was used to indicate similarity of synergies was 0.85 ± 0.03 for synergy 1, 0.89 ± 0.01 for synergy 2, and 0.87 ± 0.02 for synergy 3. The highest between-group similarity was found for the middle deltoid (0.90 ± 0.01), while the supraspinatus exhibited the lowest between-group similarity (0.52 ± 0.02) (Table 4).

**Figure 5:**
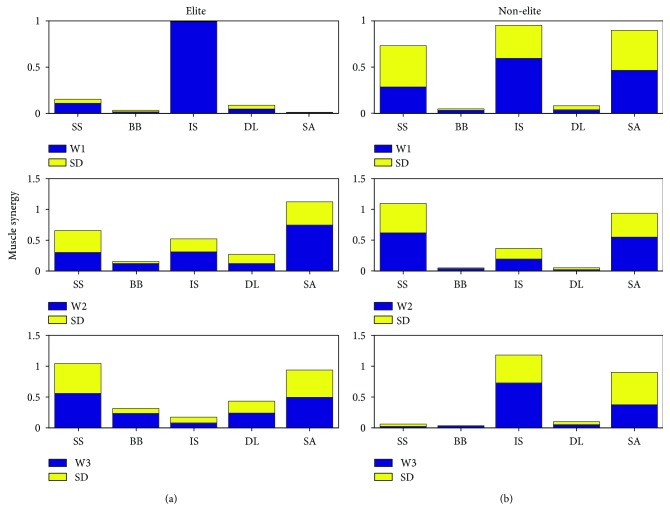
Comparison of five muscle synergies between (a) elite and (b) nonelite groups with their standard deviations.

Figure 5 represents the three muscle synergy weights (W_1_, W_2_, and W_3_) calculated from the 5 muscles of the elite and nonelite groups. Each bar chart presents activation levels of 5 upper limb muscles within each synergy weight during the BFOS. The synergy weight W_1_ of the IS muscle presents significant differences (*P* < 0.05) compared to other muscles in the elite group (W_1_ = 0.99 ± 0.004). Regarding nonelite group, the IS muscle presents differences compared to other muscles in synergy weight W_1_ (W_1_ = 0.59 ± 0.27). The synergy weight W_2_ of the SA muscle represents synergy weight W_2_ = 0.74 ± 0.35 for elite players. The synergy weight W_2_ of the SS muscle represents synergy weight W_2_ = 0.61 ± 0.35 for nonelite players. The synergy weight W_3_ of the SS and the SA muscles present differences compared to other muscles in the elite group (W_3_ = 0.55 ± 0.91 for the SS and W_3_ = 0.49 ± 0.31 for the SA). Synergy weight W_3_ for the IS muscle presents high differences compared to other muscles in the nonelite group (W_3_ = 0.72 ± 0.45).

**Figure 6:**
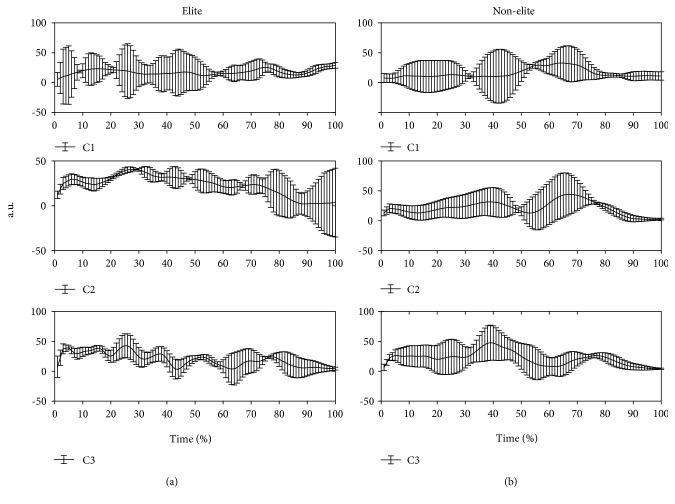
Comparison of coactivation coefficients between (a) elite and (b) nonelite groups with standard deviations.

The calculated synergies and their coactivation of synergy coefficients and time series of synergies are featured in Figure 6.

The first synergy W_1_ is mainly composed of the infraspinatus (IS) that are major contributors to the flexion and abduction motions in the elite group. Although, the first synergy W_1_ of the nonelite group, is composed of the supraspinatus (SS) and the infraspinatus (IS), along with the serratus anterior (SA) that are major contributors to the rotation and abduction movements. The second synergy of the elite group contains major weighting from the serratus anterior (SA) contributing to the upward rotation motion and is highly activated in follow-through phase of BFOS. The second synergy of the nonelite group contains weighting from the supraspinatus (SS) and the serratus anterior (SA), contributing to the abduction, and upward rotation. The third synergy of the elite group is made up of the supraspinatus (SS) and the serratus anterior (SA) responsible for shoulder abduction and upward rotation. The third synergy of the nonelite group is dominated by the activity of the infraspinatus (IS) and the serratus anterior (SA) in comparison with other muscles.

The synergy weights (W) and their activation coefficients (C) are averaged during BFOS for each group. The mean and SD values of activation coefficients during BFOS are presented for elite and nonelite groups separately.

Synergy 1 primarily engaged the IS muscle and activated early in the BFOS. Synergy 2 primarily engaged the SA muscle and activated early in the BFOS for elite players. Albeit, synergy 2 primarily engaged the SS and the SA muscles with peak activation at the time of impact for nonelite players. Synergy 3 primarily engaged the SS and the SA muscles and activated early in the BFOS for elite players. Synergy 3 primarily engaged the IS and the SA muscles with peak activation at 40% of the BFOS for nonelite players.

## 4. Discussion

Overhead stroke is a complex motion that uses the muscles of the upper extremities, the trunk, the shoulder, the elbow, the wrist, and the fingers. The skilled players hit a ball accurately, quickly, and repetitively because these muscles are well coordinated and well conditioned. The goal of our study was to examine how the muscle synergy pattern depends on the quality of players. We analyzed muscle activities and synergies in badminton forehand overhead smash movement in two different level groups (elite and nonelite) to finding how muscles activate together to produce the stroke in elite and nonelite groups. Five different upper limb muscle activities were recorded through surface EMG.

The result presents that three synergies can describe the discovered neuromuscular patterns sufficiently in elite and nonelite groups. The first synergy weight (W_1_) activated the IS muscle in elite and the IS, SS, and SA muscles in the nonelite group, the main muscles which produce an external rotator of the shoulder and abduction of the arm; however, in the nonelite group, there was also a large activation level of the IS muscle corresponding to intensive shoulder motion, which means the players used the muscles of the shoulder to control the upper arm to drive the arm forwards.

In the second synergy weight (W_2_) of the elite and non-elite athletics, there were influenced activation levels in the SS, SA, and IS muscles, which these muscles were activated for shoulder movements. Large activation level of the SA and the SS muscles was observed in the synergy weight (W_3_) in elite players and the SA and the SS muscles were observed in the nonelite group. Additionally, the SA muscle can lift the ribs when the shoulder girdle is fixed. Therefore, after the contact point, elite players utilized neuromuscular control strategies differently and strongly. However, at least we suggested that muscle synergies can explain adequately the observed EMGs in BFOS stroke. Also, nonelite players seemed to use different neuromuscular motor strategies to get the stroke. Synergy weight W_1_ includes the complex movement needed during the specific phase. Therefore, the most active muscles correspond to movement such as shoulder abduction, external rotation, and scapular retraction. This synergy was also active after contact point, as a decelerator. Synergy weight W_2_ consists of motions vital before contact phase, such as trunk extension, rotation, and elbow extension. Finally, synergy weight W_3_ consists of movements that supply before and after contact point (acceleration and deceleration phase), such as shoulder internal rotation and elbow flexion.

Synergy 1 (C_1_) was considered the same in both elite and nonelite groups. It primarily engaged the IS muscle, and peak activation was approximately 70–80% of the BFOS. The IS muscle has the function of external rotation. It was assumed that the role of synergy 1 was to rotate and abduct the arm for preparing to hit a shuttlecock at a higher point.

Synergy 2 (C_2_) was primarily engaged the IS, SS, and SA muscles, and peak activation was approximately 20–30% (for elite) and 60–70% (for nonelite) of the BFOS. About synergy coactivation coefficients, the third coefficients (C_3_) in elite and nonelite groups were similar across the subjects, whereas the first and second coefficients (C_1_ and C_2_) might be group specific. The first synergy in elite players activated and got to the peak before the contact point while the nonelite group activated at contact time percentage. The second synergy in elite players activated and got to the peak before and after the contact point while the nonelite group activated just before contact point. The first time course activations between elite and nonelite groups were different, i.e., with elite players, the first synergy activated immediately but nonelite players showed a different pattern. The variability witnessed might be caused by different postures and strategies of badminton players with different level of skill, which we will discuss later.

Muscle synergy study has the potential to give a global picture of the muscle coordination strategies [10, 29]. Overall, the two different groups represented the same number of synergies suggesting a similar complexity of motor control between elites and nonelites. Muscle synergy activation coefficients were similar between groups for synergy 1, 2, and 3.

Similar EMG patterns have been reported between elite and non-elite players during rowing [20] and bench press [13], which can be considered as less complex motor tasks than badminton forehand overhead smashes. Because badminton forehand overhead smash involves numerous degrees of freedom from upper limbs, many different motor coordination strategies are available to perform the overhead motion.

The current study includes some limitations that should be acknowledged. First, EMG signals of only five muscles were recorded for the current study and teres minor, teres major, and subscapularis EMG activities would calculate during BFOS. Using a low number of muscles has been shown to lead to poor reliability of the extracted synergies and to an artificial increase in the reconstruction accuracy. This is a potential limitation of the study. Secondly, we analyzed and compared elite and nonelite athletics, albeit in future studies, middle skill level athletics would participate in a study.

## 5. Conclusions

The present study is analyzed to understand the upper limb muscles strategies during badminton forehand overhead smash task. We have evidenced the presence of synchronous muscle synergies in a badminton forehand overhead smash and analyzed their relationship with biomechanical outputs. The results procured from the study suggested that three synergies are sufficient to explain the muscle parameters variability in EMG observed from five upper limb muscles for elite and nonelite groups. The similarities between groups are also represented. In future research, we will study muscles from the trunk and lower extremities. But, it is vital to study muscle activities of the third group between elite and nonelite players in BFOS to clarify differences for training and whether muscle synergistic patterns of them are different or similar.

## Figures and Tables

**Table 1:** Anthropometry characteristics of the participants.

	Elite badminton players	Nonelite badminton players
Age (years)	24 ± 1.7	26 ± 2.9
Height (cm)	175 ± 3.3	173 ± 2.4
Weight (kg)	71 ± 2.2	75 ± 1.6
Experience (years)	11 ± 0.8	2 ± 2.1

All values are means ± SD.

**Table 2:** Mean value of local VAF for each muscle when elite and nonelite athletics had three synergies. VAF: variance accounted for.

Muscle		VAF (mean ± SD)
Supraspinatus	Elite	0.90 ± 0.029
	Nonelite	0.91 ± 0.032

Biceps brachii	Elite	0.93 ± 0.018
	Nonelite	0.91 ± 0.026

Infraspinatus	Elite	0.95 ± 0.021
	Nonelite	0.90 ± 0.031

Middle deltoid	Elite	0.93 ± 0.011
	Nonelite	0.89 ± 0.071

Serratus anterior	Elite	0.92 ± 0.042
	Nonelite	0.94 ± 0.019

**Table 3:** Mean value of scalar product for 3 trials in each subject for elite and nonelite athletics.

		Synergy 1 ± SD	Synergy 2 ± SD	Synergy 3 ± SD
Elite	Subject 1	0.89 ± 0.04	0.83 ± 0.03	0.78 ± 0.05
	Subject 2	0.92 ± 0.01	0.86 ± 0.02	0.82 ± 0.03
	Subject 3	0.95 ± 0.03	0.89 ± 0.05	0.85 ± 0.02
	Subject 4	0.92 ± 0.05	0.85 ± 0.03	0.80 ± 0.04
	Subject 5	0.94 ± 0.02	0.88 ± 0.08	0.82 ± 0.01
	Subject 6	0.93 ± 0.08	0.87 ± 0.02	0.78 ± 0.03
	Subject 7	0.93 ± 0.01	0.89 ± 0.01	0.80 ± 0.02
	Subject 8	0.88 ± 0.04	0.81 ± 0.04	0.76 ± 0.05
	Subject 9	0.91 ± 0.01	0.88 ± 0.02	0.81 ± 0.03
	Subject 10	0.92 ± 0.02	0.89 ± 0.03	0.82 ± 0.01
	Subject 11	0.93 ± 0.03	0.87 ± 0.02	0.83 ± 0.02
	Subject 12	0.89 ± 0.01	0.80 ± 0.06	0.77 ± 0.06

Nonelite	Subject 13	0.89 ± 0.04	0.82 ± 0.05	0.76 ± 0.03
	Subject 14	0.91 ± 0.01	0.84 ± 0.02	0.79 ± 0.01
	Subject 15	0.92 ± 0.01	0.88 ± 0.03	0.81 ± 0.02
	Subject 16	0.95 ± 0.09	0.89 ± 0.02	0.83 ± 0.01
	Subject 17	0.90 ± 0.01	0.85 ± 0.02	0.80 ± 0.05
	Subject 18	0.92 ± 0.04	0.89 ± 0.01	0.82 ± 0.01
	Subject 19	0.91 ± 0.01	0.87 ± 0.03	0.81 ± 0.04
	Subject 20	0.96 ± 0.06	0.89 ± 0.04	0.85 ± 0.02

**Table 4:** Mean value of scalar product of muscles and synergies for elite and non-elite athletics.

		Scalar product ± SD
Muscle	Supraspinatus	0.52 ± 0.02
	Biceps brachii	0.78 ± 0.01
	Infraspinatus	0.70 ± 0.03
	Middle deltoid	0.90 ± 0.01
	Serratus anterior	0.82 ± 0.02

Synergy	Synergy 1	0.85 ± 0.03
	Synergy 2	0.89 ± 0.01
	Synergy 3	0.87 ± 0.02

## Data Availability

The data used to support the findings of this study are available from the corresponding author upon request.
